# The plasma viral communities associate with clinical profiles in a large-scale haematological patients cohort

**DOI:** 10.1186/s40168-024-01855-4

**Published:** 2024-07-23

**Authors:** Shuai Ma, Yuyao Yin, Yifan Guo, Chaoqun Yao, Siqi Xu, Qingqing Luo, Guankun Yin, Shuyi Wang, Qi Wang, Hongbin Chen, Ruobing Wang, Longyang Jin, Guanxiang Liang, Hui Wang

**Affiliations:** 1 Department of Clinical Laboratory, Peking University People’s Hospital, Beijing, 100044 China; 2 Institute of Medical Technology, Peking University Health Science Center, Beijing, China; 3 Center for Infectious Disease Research, School of Medicine, Tsinghua University, Beijing, China; 4 Tsinghua-Peking Center for Life Sciences, Beijing, China

**Keywords:** Haematologic disorders, Metagenomic sequencing, Plasma Virome, TTV

## Abstract

**Background:**

Haematological patients exhibit immune system abnormalities that make them susceptible to viral infections. Understanding the relationship between the virome in the blood plasma of haematological patients and their clinical characteristic is crucial for disease management. We aimed to explore the presence of viral pathogens and identify close associations between viral infections and various clinical features.

**Results:**

A total of 21 DNA viruses and 6 RNA viruses from 12 virus families were identified from 1383 patients. Patients with haematological diseases exhibited significantly higher diversity, prevalence, and co-detection rates of viral pathogens. During fever episodes, pathogen detection was notably higher, with Epstein-Barr virus (EBV) and Mucorales infections being the most probable culprits for fever symptoms in non-haematological patients. The detection rate of torque teno virus (TTV) significantly increases in haematological patients after transplantation and during primary lung infections. Additionally, TTV-positive patients demonstrate significantly higher absolute neutrophil counts, while C-reactive protein and procalcitonin levels are notably lower. Furthermore, TTV, cytomegalovirus, and parvovirus B19 (B19V) were found to be more prevalent in non-neutropenic patients, while non-viral pathogenic infections, such as Gram-negative bacteria and Mucorales, were more common in neutropenic patients. Pegivirus C (HPgV-C) infection often occurred post-transplantation, regardless of neutropenia. Additionally, some viruses such as TTV, B19V, EBV, and HPgV-C showed preferences for age and seasonal infections.

**Conclusions:**

Analysis of the plasma virome revealed the susceptibility of haematological patients to plasma viral infections at specific disease stages, along with the occurrence of mixed infections with non-viral pathogens. Close associations were observed between the plasma virome and various clinical characteristics, as well as clinical detection parameters. Understanding plasma virome aids in auxiliary clinical diagnosis and treatment, enabling early prevention to reduce infection rates in patients and improve their quality of life.

Video Abstract

**Supplementary Information:**

The online version contains supplementary material available at 10.1186/s40168-024-01855-4.

## Background

Patients with haematological disorders frequently experience weakened immune systems due to their underlying diseases or treatments, making them susceptible to various infections, increasing the complexity of their treatment, and affecting their survival rates [1]. Primary bloodborne viral infections and reactivation are common among haematological patients, particularly those undergoing allogeneic haematopoietic stem cell transplantation (allo-HSCT), with significant incidence and mortality rates [2–4]. A better understanding of the relationship between viral composition in haematological patients and disease progression is crucial.

Advancements in metagenomic next-generation sequencing (mNGS) have provided insights into patient viromes; however, research has primarily focused on the gastrointestinal and respiratory tracts [5–7]. Additionally, studies investigating the plasma viromes of patients undergoing solid organ transplantation (SOT) and allo-HSCT have revealed the virome landscape of transplant recipients [8–12]. In clinical practice, most studies focus on viruses that can cause severe infections or life-threatening diseases, such as severe acute respiratory syndrome coronavirus 2 (SARS-CoV-2), influenza virus, and norovirus, among others [5–7]. Numerous viruses, such as torque teno tirus (TTV), human pegivirus C (HPgV-C), and human herpesvirus 6 (HHV-6), are frequently detected but often manifest as asymptomatic infections [12]. These asymptomatic viral infections are prevalent in humans; however, their role in disease progression remains unknown.

In healthy individuals, a diverse range of viral colonisation exists [13, 14]. This includes latent infections in the blood by viruses such as TTV, herpes simplex virus (HSV), Epstein-Barr virus (EBV), cytomegalovirus (CMV), and HPgV-C; the abundant presence of bacteriophages in the gut, along with certain eukaryotic viruses; and commonly detected viruses in the respiratory tract, such as human rhinovirus and respiratory syncytial virus [5, 12, 15, 16]. The functions of these asymptomatic viral infections are currently being studied. For example, exposure to rhinovirus, the predominant etiological agent of the common cold, confers protection against SARS-CoV-2, the causative agent of the coronavirus disease 2019 [17–19]. Increased picornavirus levels in the gastrointestinal tract can serve as predictive markers for the development of gastrointestinal graft-versus-host disease in patients undergoing HSCT [20]. Additionally, blood-borne HPgV-C infection exerts a certain degree of inhibitory effect during the progression of HIV infection [21, 22].

Emerging evidence suggests that these commensal viruses play a role in haematologic disease progression [12]. The presence of TTV in haematological patients can serve as an indicator for monitoring the status of the immune system and potential infection risks [23]. Infections caused by members of the Herpesviridae family may lead to a range of clinical symptoms and complications, such as rashes and pneumonia [24]. Additionally, human parvovirus B19 (B19V) infection can result in severe anaemia and other clinical manifestations, particularly in cases where the immune system is compromised. Some members of the polyomaviridae family, such as human polyomavirus 1 (HPyV-1), are associated with haemorrhagic cystitis in haematologic patients, while the reactivation of human polyomavirus 2 (HPyV-2) may lead to the development of neurological diseases, such as progressive multifocal leukoencephalopathy [25–27].

Although some progress has been made, there is currently a lack of large-scale studies involving a comprehensive analysis of the plasma virome in haematological patients in relation to various clinical characteristics. In this study, we recruited a cohort of 1382 patients, including 849 haematologic and 534 non-haematologic patients. We conducted metagenomic analyses to explore the presence of viral pathogens. Additionally, we integrated virome data with relevant clinical data, and identified close associations between viral infections and various clinical features.

## Methods

### Study patients and samples

**Fig. 1:**
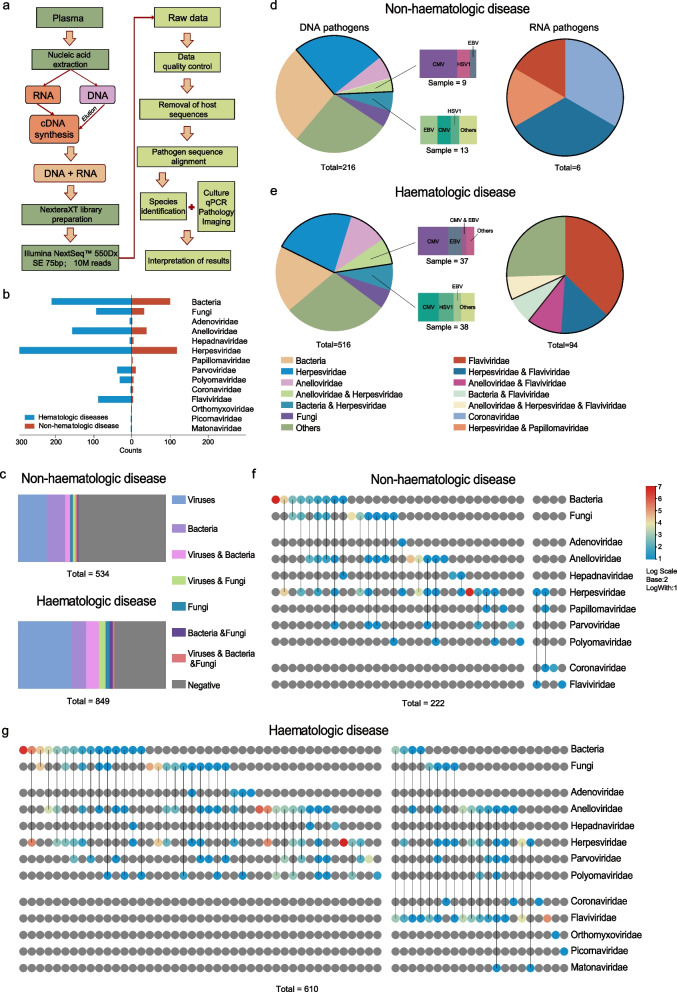
The viral composition, pathogen diversity, and co-infection analysis of plasma samples from patients. **a** Flowchart for pathogen detection in plasma samples by metagenomic next-generation sequencing (mNGS). **b** Total number of pathogens detected in patients with haematological and non-haematological diseases. The viruses were categorised according to their families. **c** Composition ratio of bacterial, fungal, and viral or mixed infections in patients with haematological and non-haematological diseases. **d**, **e** Composition ratio of uniquely detected positive single pathogen and mixed infection pathogen combinations in patients with non-haematologic or haematologic diseases. The left panel depicts the overall composition ratio of DNA pathogens, specifically the composition ratio of species from the Herpesviridae family co-infected with Anelloviridae and bacteria. The right panel shows only the composition ratio of RNA pathogens categorised by viral family. **f**, **g** The *x*-axis represents uniquely detected positive single pathogens and mixed infection pathogen combinations in non-haematological or haematological patients, with multiple pathogens in mixed infections connected by lines. The number of patients with mixed infectious pathogen combinations is represented by colour depth, with grey indicating no infection by this pathogen, in proportions represented on a logarithmic scale with a base of 10. The viruses were categorised according to their families

This study collected clinical information from 1601 patient samples that underwent plasma mNGS testing in the Department of Laboratory Medicine at Peking University People’s Hospital from April 1, 2022, to August 31, 2023 (Supplementary Table  sheet 1). After excluding patient samples submitted within 1 month of each other, a total of 1383 cases were included in the analysis. Informed consent was obtained from all participants in the study. Clinical data, including patient white blood cell count (WBC), absolute neutrophil count (ANC), absolute lymphocyte count (ALC), red blood cell count (RBC), haemoglobin (HGB), C-reactive protein (CRP), and procalcitonin (PCT), as well as information regarding patients’ underlying medical conditions and infection sites, were collected simultaneously with mNGS testing (Fig. 1a). Given our primary focus on the detection of viral pathogens, non-viral pathogens were not further classified to the species level, and the statistics for RNA virus detection were completed within the samples processed through the DNA and RNA workflow.

### Profiles of 1383 patients with clinical features and pathogen detection

Plasma mNGS testing was divided into the DNA, DNA and RNA workflows. We excluded samples from the analysis that were either submitted for testing within 1 month or lacked any clinical information. This left us with a total of 1383 plasma mNGS samples for analysis. The samples were evenly distributed throughout the year, with a median patient age of 50 years (interquartile range IQRs 30, 63). Among these samples, 800 (57.84%) were from male patients, and 849 (61.39%) were from patients with haematologic conditions originating from three different hospital branches.

The clinical characteristics of the patients included information such as the onset of symptoms, underlying conditions, infection site, date of pathogen detection, and clinical biomarkers. Underlying conditions were determined according to the World Health Organization definitions and included various chronic diseases affecting different organs, such as diabetes, solid tumours, hyperlipidaemia, and autoimmune diseases. The infection site specifically refers to infections caused by non-viral pathogens. The infection site was determined based on clinical diagnosis and other clinical pathogen detection information, considering data from 3 days before and after the sample collection date. Clinical biomarkers were obtained from various clinical tests such as complete blood counts and biochemical profiles. Except for CRP and PCT, which utilised data from 3 days before and after the recorded detection, other biomarkers were derived from the same-day test results (Table 1).

**Table 1:** Patient demographics and clinical features

Characteristic	Haematological diseases	Non-haematological diseases
	Value (median (IQRs) or no. (%))	
Total sample size	849	534
Age (years)	42 (22, 58)	59 (41, 71)
Distribution		
0–18 years old	182 (21.44%)	27(5.06%)
19–45 years old	275 (32.39%)	126 (23.60%)
46–65 years old	300 (35.34%)	186 (34.83%)
> 65 years old	91 (10.72%)	188 (35.21%)
Male sex	515(60.66%)	285 (53.37%)
Branch hospital 1	398(46.88%)	436(81.65%)
Branch hospital 2	325 (38.28%)	72(13.48%)
Branch hospital 3	126 (14.84%)	26(4.87%)
Onset symptoms		
HSCT/SOT	370 (43.58%)	28(5.24%)
Neutropenia	370 (43.58%)	0
Fever	514 (60.54%)	375(70.22%)
COVID-19	26 (3.06%)	49(9.18%)
Underlying condition		
Cardiovascular disease	77 (9.07%)	181 (33.90%)
Lung disease	26 (3.06%)	61 (11.42%)
Kidney disease	17 (2.00%)	68 (12.73%)
Liver disease	45 (5.30%)	98 (18.35%)
Solid tumour	32 (3.77%)	64 (11.99%)
Autoimmune disease	24 (2.83%)	71 (13.30%)
Diabetes	39 (4.59%)	73 (13.67%)
Hyperlipidemia	17 (2.00%)	27 (5.06%)
Infection site		
Bloodstream infection	33 (3.89%)	50 (9.36%)
Pulmonary infection	154 (18.14%)	155 (29.03%)
Urinary tract infection	13 (1.53%)	16 (3.00%)
Skin and Soft Tissue Infection	10 (1.18%)	5 (0.94%)
Central nervous system infection	3 (0.35%)	0
Gastrointestinal infection	13 (1.53%)	10 (1.87%)
Sample collection time		
Mar to May	253 (29.80%)	126 (23.60%)
Jun to Aug	350 (41.22%)	313 (58.61%)
Sep to Nov	121 (14.25%)	37 (6.93%)
Dec to Feb	125 (14.72%)	58 (10.86%)
Clinical biomarker		
WBC (10^9^/L)	2.06 (0.47, 4.8)	7.33 (5.3, 10.83)
< 4	589 (69.38%)	34 (6.37%)
4–10	185 (21.79%)	308 (57.68%)
> 10	73 (8.60%)	147 (27.53%)
ANC (10^9^/L)	0.81 (0.05, 2.77)	5.49 (2.29, 8.99)
< 1.8	538 (63.37%)	41 (7.68%)
1.8–6.3	223 (26.27%)	258 (48.31%)
> 6.3	79 (9.31%)	219 (41.01%)
ALC (10^9^/L)	0.41(0.14, 0.88)	0.99 (0.61, 1.49)
< 0.8	611 (71.97%)	198 (37.08%)
0.8–4	211 (24.85%)	314 (58.80%)
> 4	19 (2.24%)	6 (1.12%)
RBC (10^12^/L)	2.18 (1.84, 2.67)	3.53 (2.98, 4.07)
< 3.8	814 (95.88%)	319 (59.74%)
3.8–5.1	26 (3.06%)	181 (33.90%)
> 5.1	2 (0.24%)	9 (1.69%)
HBG (g/L)	69 (58,83)	107 (89, 122)
< 115	812 (95.64%)	320 (59.93%)
115–150	30 (3.53%)	179 (33.52%)
> 150	0	10 (1.87%)
CRP (mg/L)	58.2 (17.9, 115.4)	41 (13.52, 90.08)
< 10	152 (17.90%)	98 (18.35%)
10–50	225 (29.50%)	169 (31.65%)
> 50	464 (54.65%)	217 (40.64%)
PCT (ng/mL)	0.230 (0.106, 0.627)	0.196 (0.071, 0.764)
≤ 0.5	477 (56.18%)	322 (60.30%)
> 0.5	211 (24.85%)	139 (26.03%)
TBil (umol/L)	12.3 (8.4, 19.98)	9.5 (6.2, 17.1)
< 3	13 (1.53%)	15 (2.81%)
3–21	604 (71.14%)	371 (69.48%)
> 21	177 (20.85%)	95 (17.79%)
Testing method		
DNA detection	350 (41.23%)	317 (59.36%)
DNA&RNA detection	499 (58.78%)	217 (40.64%)
Pathogen detection rate	554 (65.25%)	219 (41.01%)
Distribution		
Virus	424 (49.94%)	138 (25.84%)
Bacteria	190 (22.38%)	95 (17.79%)
Fungi	90 (10.60%)	31 (5.81%)
Total sample size	849	534

*Abbreviations:* *WBC* white blood cells, *ANC* absolute neutrophil count, *ALC* absolute lymphocyte count, *RBC* red blood cell, *HBG* haemoglobin, *CRP* C reactive protein, *PCT* procalcitonin, *TBil* total bilirubin

### Plasma sample processing and viral nucleic acid extraction

Plasma samples were obtained from EDTA anticoagulated whole blood by centrifugation at 3500 rpm for 10 min. Nucleic acid extraction was carried out through two distinct procedures: the DNA extraction protocol (*N* = 667) and the DNA and RNA extraction protocol (*N* = 716). In the DNA extraction protocol, the PathoXtract WYXM500S mcfDNA Enrichment and Extraction Kit (WillingMed, Beijing, China) was used to extract nucleic acids from 200 μL of plasma. The extracted nucleic acids were subsequently used for further processing [28]. For the DNA and RNA extraction protocol, DNA and RNA were extracted separately, following the same initial steps and the same kit as the DNA extraction protocol. The extracted RNA nucleic acid was reverse-transcribed using HiScript III All-in-one RT SuperMix (Vazyme, Nanjing, China), and the resulting cDNA was purified using Agincourt AMPure XP magnetic beads. In the final elution step, using the DNA extracted in the previous step to elute cDNA helps reduce the elution volume, increase nucleic acid concentration, facilitate library construction, and achieve simultaneous detection of both DNA and RNA (Fig. 1a).

### Sequencing library preparation and bioinformatics analysis

NGS library preparation was carried out using the Illumina® DNA Prep (M) Tagmentation kit (20,018,705; Illumina, San Diego, USA). For each sequencing run, a negative control (NC) was included. In total, 200 sequencing runs were performed. The libraries were quantified using the Qubit dsDNA HS Assay Kit (Thermo Fisher Scientific, Waltham, MA, USA), and subsequently, they were pooled together in equimolar concentrations. The sequencing was performed on the NextSeq™ 550Dx sequencer (Illumina, San Diego, USA) with 75 bp single-end reads, using a high-output kit, yielding a minimum of 20 million sequencing reads per sample. The raw sequencing data was filtered using Trimmomatic (v0.40) to remove low-quality sequences. The quality settings used for Trimmomatic filtering were as follows: ILLUMINACLIP:TruSeq3-SE:2:30:10, SLIDINGWINDOW:4:20, and MINLEN:50. After filtering, the processed data was aligned against the human reference genome GRCH38 with Bowtie 2 (v2.2.2) to exclude human-related sequences. Subsequently, the remaining sequences were matched against a pre-constructed reference database using Kraken2 (v2.1.0) to detect potential pathogens within the samples. The reference database used for Kraken2 analysis was PlusPF database (downloaded on 9/12/2022), which contains refseq archaea, bacteria, viral, fungi, and protozoa. The identified pathogen sequences were standardised to reads per million (RPM) values. To determine positive results, the RPM-ratio was calculated by comparing against a concurrently processed negative control [28].

### Pathogen detection thresholds

Distinct positive thresholds were established to rule out potential contamination during the experimental process and to mitigate the occurrence of false-positive results. These thresholds were determined based on clinical outcomes from other microbial tests and physiological markers, and were utilised to ascertain the presence of pathogens within the samples. Comprehensive clinical reports were generated for each patient. For bacteria and fungi, the positive threshold was defined as RPM-ratio ≥ 10. However, for tuberculosis or nontuberculous mycobacteria (TB/NTM) and rare pathogens, the positive threshold was defined as reads ≥ 1. Concerning viruses, the positive threshold was set as the presence of reads spanning at least 3 distinct non-overlapping regions of the genome, and reads ≥ 3 [29–33].

### Statistical analysis

Following a normality test, the data in this study was found to be non-normally distributed. Non-parametric statistical tests, including the Mann–Whitney *U* test, were used to characterise the probability of data in the correlation analysis. To compare differences in disease groups using pathogen detection rate, the pathogen detection rate is calculated by dividing the number of individuals detected with the pathogen by the total sample size, then multiplying by 100%. Categorical data were analysed using the chi-square test. Statistical analyses were performed using GraphPad Prism version 9.0.2 (San Diego, CA, USA). To analyse the factors associated with pathogen detection, we established a logistic regression model using SPSS version 26.0 (IBM Corp, Armonk, NY), and odds ratios (OR) were calculated. All models successfully passed the omnibus test and the Hosmer–Lemeshow goodness-of-fit test.

## Results

### Pathogen co-detection frequently occurs in haematologic patients

The detection rates for bacteria and fungi were 20.61% and 8.75%, respectively. The most frequently identified viral families were Herpesviridae (25.87%; mainly CMV, EBV, HHV-6B, and HSV-1), Anelloviridae (14.03%; mainly TTV), Parvoviridae (3.47%; mainly B19V), and Flaviviridae (12.70%; mainly included HPgV-C). The results of the chi-square test indicated that among the DNA viruses detected, the overall viral detection rate was higher in haematologic patients than in non-haematologic patients (43.93 vs 25.47%, *P* < 0.05, *χ*^2^ = 22.49). For RNA viruses detected, the detection rate of Flaviviridae was significantly higher in haematologic patients and was statistically significant (17.43 vs 0.92%, *P* < 0.0001, *χ*^2^ = 30.07; Fig. 1b). Overall, haematologic patients exhibited significantly higher detection rates, then the non-haematologic disease group (65.25 vs 41.01%, *P* < 0.05, *χ*^2^ = 20.61; Fig. 1c, Supplementary Table sheet 2).

Prior research has documented the concurrent detection of multiple pathogens in hospitalised patients [34]. A comprehensive examination of the overall landscape of pathogen co-detection in haematological patients was conducted using our extensive cohort. We identified 335 co-infection events in which at least two types of pathogens were detected in the same patient. Additionally, 497 patients were infected with only one type of pathogen. Notably, when compared to the non-haematologic disease group, the haematologic disease group exhibited a significantly higher ratio of co-infection events (48.38 vs 30.59%, *P* < 0.05, *χ*^2^ = 8.042; Fig. 1d, e, Supplementary Table sheet 2).

Co-infection with bacterial and viral pathogens was prevalent. For example, the co-detection of Herpesviridae and bacteria was one of the most frequent co-detection events (*N* = 75). Notably, our primary focus in this analysis was virus detection, and we did not conduct an in-depth examination of the simultaneous detection of bacterial and fungal pathogens.

Among the viral families, co-infection with Herpesviridae and Anelloviridae was the most common (*N* = 78). Herpesviridae co-infections with bacteria and Anelloviridae primarily include CMV, EBV, and HSV-1. Additionally, the detection rate of RNA viruses in the haematologic disease group was significantly higher than that in the non-haematologic disease group, which was primarily attributed to Flaviviridae infection. In haematologic patients, similar to DNA viruses, co-infections involving RNA viruses mainly included the simultaneous detection of Flaviviridae, Herpesviridae, Anelloviridae, and bacteria (Fig. 1d, e). Overall, the combination of pathogens detected in patients with haematologic disease was more diverse than that in patients without haematologic disease (Fig. 1f, g).

### Relationship between viral infections and fever in haematologic patients

**Fig. 2:**
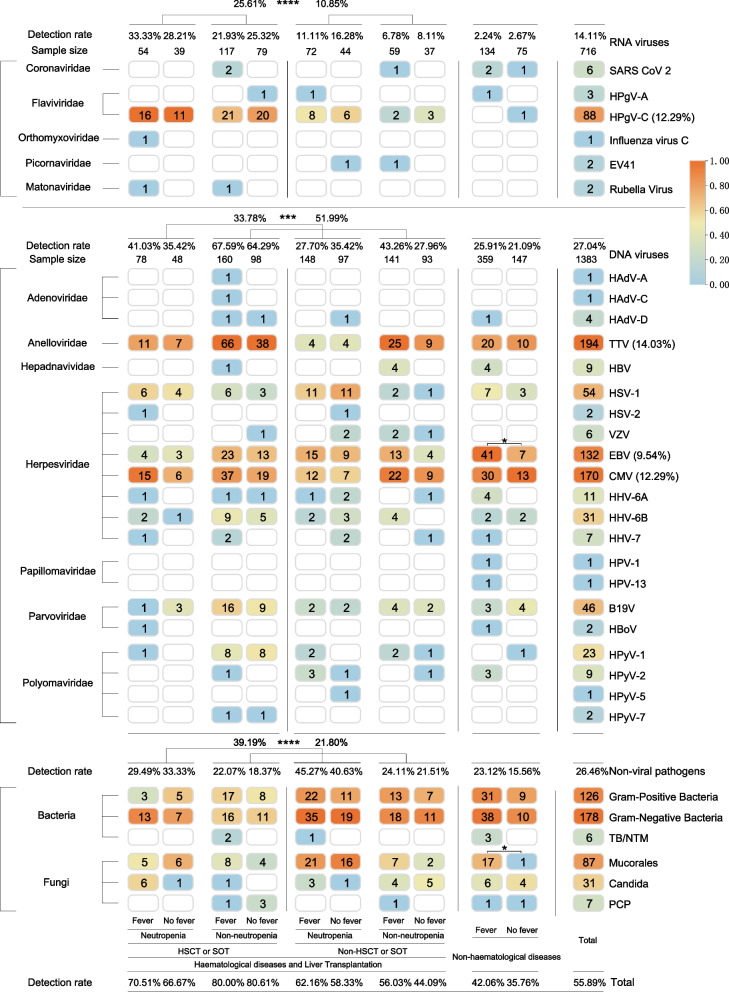
Comparative analysis of fever presentation and pathogen detection in haematologic and non-haematologic patient categories. Detection status of the 1383 patient samples based on fever presentation. The patients were categorised into haematologic and non-haematologic disease groups. Within the haematologic disease group, patients were further divided into the transplant with neutropenia, transplant without neutropenia, non-transplant with neutropenia, and non-transplant without neutropenia groups. Each group was compared based on the presence or absence of fever. The numbers in the figure indicate the sample sizes, number of detections, detection rates for each pathogen class, total sample size, and overall detection rate for each category. The heatmap was subjected to column standardisation, and a log10 transformation was applied only to the colours of the heatmap; numerical values were not transformed. Note: The focus of this figure is the detection differences of individual viruses among different groups. Due to the possibility of a single patient being infected with multiple pathogens, the detection rate in the figure is not simply calculated by adding and dividing by the total number. Please refer to the Supplementary Table for the specific infection status of each patient.*P < 0.05, **P < 0.01, ***P < 0.001, ****P < 0.0001

Fever with an unknown cause is a serious symptom in haematologic patients [35]; therefore, we conducted an analysis to explore its association with pathogenic infections. We divided the patients into the following groups: HSCT or SOT, non-HSCT or SOT, and non-haematological disease groups. Within the haematologic disease group, we further subdivided the patients into neutropenia and non-neutropenia subgroups (Fig. 2).

The most frequently detected viruses were TTV (14.03%), CMV (12.29%), EBV (9.54%), and HPgV-C (12.29%). The overall detection rate of DNA viruses was higher in the non-neutropenia group (51.99 vs 33.78%, *P* < 0.01, *χ*^2^ = 11.11; Fig. 2), whereas the detection rate of non-viral pathogens was higher in the neutropenia group (39.19 vs 21.80%, *P* < 0.01, *χ*^2^ = 16.35; Fig. 2). The detection rates of both pathogens in the non-haematological disease group were lower than those in the haematological disease group. Additionally, we observed that among DNA viruses, TTV, CMV, B19V, and HPyV-1 had higher detection rates in the non-HSCT or SOT patients in the non-neutropenia group, albeit without statistical significance, while HPgV-C infection among RNA viruses was more common in the HSCT or SOT group (25.61 vs 10.85%, *P* < 0.01, *χ*^2^ = 11.02; Supplementary Table sheet 2).

Among the 1383 patients, the pathogen detection rate in the fever group was significantly higher than that in the non-fever group (74.36 vs 53.94%, *P* < 0.05, *χ*^2^ = 10.61; Supplementary Table sheet 2). In the haematologic disease group, the detection rates of bacteria, DNA viruses, such as TTV, EBV, CMV, and HHV-6B, and RNA viruses, such as SARS-CoV-2, influenza virus C, and rubella virus, were higher in the fever group then in the non-fever group, although the results were not statistically significant. Conversely, in the non-haematological disease group, EBV and Mucorales were detected at significantly higher rates in the fever group than in the non-fever group (*P* < 0.05; Fig. 2).

### Virome profiles in haematological patients associated with neutropenia

In clinical practice, patients with haematological disorders and neutropenia often receive significant attention when experiencing infections [36]. Therefore, we analysed the viral signatures in patients with haematological disorders and neutropenia, haematological disorders without neutropenia, and non-haematological disorders.

**Fig. 3:**
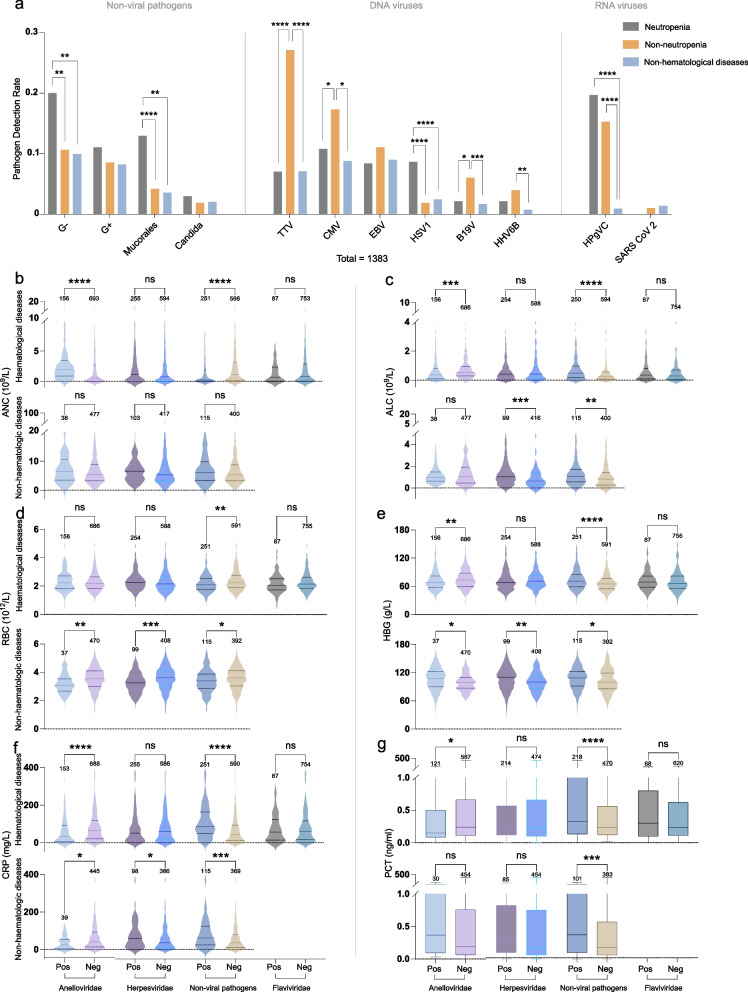
Comparative analysis of pathogen detection rates and clinical parameters in haematologic and non-haematologic patients. **a** Comparison of detection rates of frequently detected pathogens among different groups: The figure displays the differences in detection rates among the most frequently detected pathogens across the three groups: haematologic diseases with neutropenia, haematologic diseases without neutropenia, and non-haematologic diseases. Statistical analyses were performed using the chi-square test. **b**–**g** For both the haematologic and non-haematologic disease groups, non-parametric tests were conducted to compare the absolute neutrophil Count (ANC), absolute lymphocyte count (ALC), red blood cell (RBC) count, haemoglobin (HGB) level, C-reactive protein (CRP), and procalcitonin (PCT) based on the presence or absence of virus detection. Statistical significance was set at *P* < 0.05. *P < 0.05, **P < 0.01, ***P < 0.001, ****P < 0.0001. Note: G+ stands for Gram-Positive Bacteria, and G- stands for Gram-Negative Bacteria

Gram-negative bacteria and Mucorales were more prevalent in patients with neutropenia than in other groups (Fig. 3a). TTV, CMV, HHV-6B, and B19V infections were more prevalent in patients without neutropenia, whereas HSV-1 and non-viral pathogenic infections were more common in patients with neutropenia. HPgV-C infection was high in both neutropenic and non-neutropenic groups (Fig. 3a). We did not observe any differences in infections with other viruses.

To explore the relationship between these pathogens and blood cell and infection indicators, we examined the ANC, ALC, HBG, and CRP levels in infected and non-infected patients for Anelloviridae, Herpesviridae, Flaviviridae, and non-viral pathogens.

In patients with haematological disorders, we observed a significant increase in ANC in the Anelloviridae-positive group compared to the negative group, while ALC, HBG, CRP, and PCT levels were significantly lower (*P* < 0.05; Fig. 3b, c, e–g). Conversely, in patients with non-haematological disease, the Anelloviridae-positive group showed significantly higher HBG levels and significantly lower CRP and RBC levels (*P* < 0.05; Fig. 3b, c, e–g). Regarding herpesviridae infection, no significant differences were observed between groups in patients with haematological disease. Conversely, in patients without haematological disease, the positive group exhibited significant increases in ALC, HBG, and CRP levels and a significant decrease in RBC levels (*P* < 0.05; Fig. 3c–f). Among patients with non-viral pathogens, the positive group of patients with haematological disease showed significant increases in ALC, HBG, CRP, and PCT levels, whereas ANC and RBC counts were significantly lower (*P* < 0.05; Fig. 3b–g). In patients without haematological disease, the positive group showed significant increases in ALC, HBG, CRP, and PCT levels, while the RBC count was significantly lower (*P* < 0.05; Fig. 3b–g). Additionally, no significant differences in Flaviviridae infection were observed (Fig. 3b–g).

### Associations between viral infections and binary classification clinical factors

The occurrence and development of pathogenic infections are associated with several factors. For example, lung diseases can easily lead to bacterial pneumonia, and haematological diseases can trigger CMV and EBV infections; however, knowledge regarding factors related to other blood-borne viral infections remains limited [1]. Therefore, we conducted multifactorial analyses of patient information, including sex, symptom onset, haematological diseases, underlying conditions, and infection site, using logistic regression analysis to assess the detection of several prevalent pathogens.

The results showed that Flaviviridae and fungal infections were correlated with neutropenia (*P* < 0.05). Infections with Anelloviridae, Herpesviridae, Parvoviridae, and Flaviviridae were significantly associated with transplantation (*P* < 0.01). Moreover, Herpesviridae and bacterial infections were significantly associated with fever (*P* < 0.05). Infections with Anelloviridae, Herpesviridae, Polyomaviridae, and Flaviviridae were associated with the occurrence of haematological diseases (*P* < 0.05). These findings are consistent with previous conclusions [11].

**Fig. 4:**
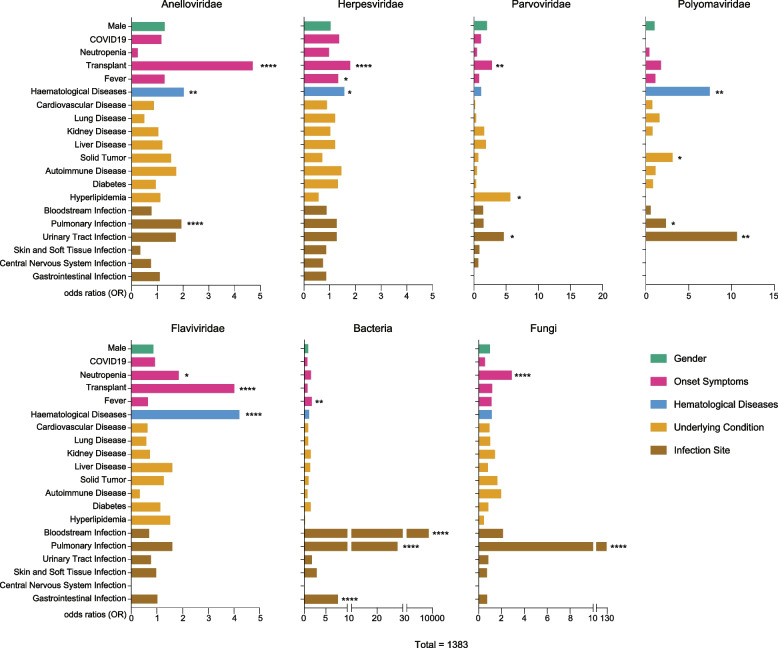
Multifactorial analysis of pathogen detection rates. A logistic regression model was used to analyse factors related to pathogen detection. Horizontal bars represent odds ratios (OR). The analysis was categorised according to the viral family. *P < 0.05, **P < 0.01, ***P < 0.001, ****P < 0.0001

Additionally, we observed that patients with hyperlipidaemia were more prone to Parvoviridae infections (*P* < 0.05), whereas patients with solid tumours were more susceptible to Polyomaviridae infections (*P* < 0.01). Considering the infection site, pulmonary infection was associated with a higher likelihood of non-viral pathogen infections (*P* < 0.0001), and Anelloviridae and Polyomaviridae were frequently detected in cases of pulmonary infection (*P* < 0.05). Moreover, the detection rate of bacteria in the bloodstream was significantly higher in patients with bloodstream and gastrointestinal infections (*P* < 0.0001). Parvoviridae and Polyomaviridae infections were more common in cases of urinary tract infection (*P* < 0.05; Fig. 4). This association remained statistically significant after adjusting for age, season, and hospital branch covariates.

### Associations between viral infections and non-binary clinical variables

To explore the differences in viral detection among patients under different conditions, we conducted statistical analyses on all samples based on different age groups, seasons, transplantation stages, and environments in which the patients were situated.

**Fig. 5:**
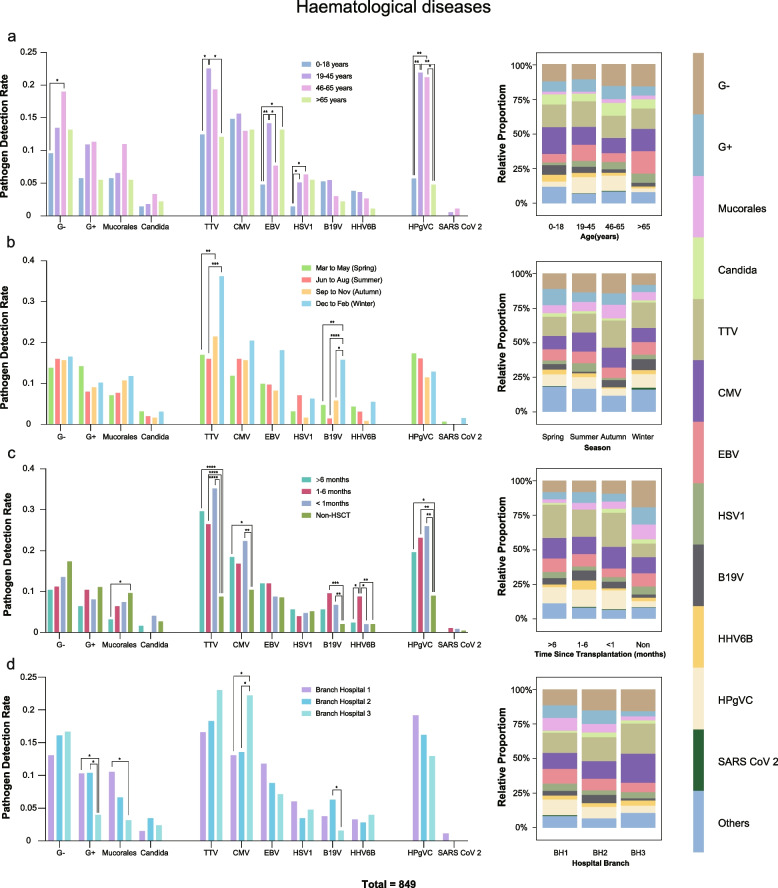
Comparative analysis of pathogen detection rates and composition ratios under various factors among haematological patients. Haematological patients were categorised according to age, season, post-transplantation time, and environment. The chi-square test was used for statistical analysis to compare differences in detection rates among the most commonly detected pathogens within each group. The composition ratios of pathogens within each subgroup are presented on the right-hand side. Statistical significance was set at *P* < 0.05. *P < 0.05, **P < 0.01, ***P < 0.001, ****P < 0.0001. Note: G+ stands for Gram-Positive Bacteria, and G- stands for Gram-Negative Bacteria

In the age group analysis, we observed that TTV, HPgV-C, and non-viral pathogens were more likely to infect individuals aged 19–45 and 45–65 among haematologic diseases. EBV is more likely to occur in individuals aged 19–45 and > 65 years. HSV1 was less common in the 0–18 years age group (*P* < 0.05; Fig. 5a) than in the 19–45 and 46–65 age groups. Only EBV had a significantly higher detection rate in non-haematological patients aged > 65 years (*P* < 0.05; Supplementary Fig. 1a).

In the seasonal group analysis, we found that TTV and B19V had higher detection rates in winter among patients with haematological diseases (Fig. 5b). Similarly, in the non-haematologic disease group, we observed a higher detection rate of TTV in winter (*P* < 0.05; Supplementary Fig. 1b). Subsequently, we grouped patients that underwent transplants and observed that TTV, CMV, B19V, and HPgV-C had significantly higher detection rates in these patients that in non-transplant patients with haematological diseases (*P* < 0.05). However, non-viral pathogens have relatively low detection rates in patients undergoing transplantation. Additionally, HHV6B had a higher detection rate in the 1–6 months post-transplantation period than in the other groups (*P* < 0.05; Fig. 5c).

Because samples of patients with haematologic disease were obtained from three different branch hospitals, we conducted subgroup analysis based on samples from different hospital branches. We found that CMV and B19V had higher detection rates in branch hospitals 3 and 2, respectively (*P* < 0.05). In contrast, G + and Mucorales had lower detection rates in Branch Hospital 3 (*P* < 0.05; Fig. 5d).

## Discussion

In this study, we performed a comprehensive analysis of plasma virome in 849 haematological and 534 non-haematological patients who underwent mNGS. To the best of our knowledge, this is the largest cohort study on plasma virome levels in patients with haematological diseases. We delved deeper into unexplored clues related to the clinical condition within the plasma viral composition of haematologic patients, thereby enhancing our understanding of plasma viral composition in this patient population.

We identified various DNA and RNA viruses, along with diverse non-viral pathogens in this cohort. Notably, the Herpesviridae, Anelloviridae, Flaviviridae, fungi, and bacteria were among the most frequently detected pathogens, consistent with prior research [11, 12, 37]. Co-detection was common, particularly involving Herpesviridae family members with bacteria or Herpesviridae family members with Anelloviridae. Haematological patients exhibited significantly higher diversity in the detected viruses, pathogen detection rates, and combinations of co-detected pathogens then non-haematologic patients. In all hospitalised patients, the detection rate of pathogens during fever episodes was significantly higher. Among non-haematologic patients, EBV and Mucorales infections were most likely to cause fever symptoms, warranting additional clinical attention. In patients with haematological disorders, there was no significant difference in the detection rate of individual pathogens between fever and non-fever episodes, which may be attributed to the higher prevalence of non-infectious fever in these patients, possibly because of regular medication regimens.

TTV is the most common virus found in the plasma of haematological patients, with increased viral load being prevalent post-transplantation. TTV often co-infects Herpesviridae and Flaviviridae, and its presence is not directly associated with fever symptoms in patients. Compared to non-haematological patients and patients with granulocytopenia, the detection rate of TTV was significantly higher in non-neutropenic haematological patients, possibly because TTV targets peripheral blood mononuclear cells and replicates within them [38–40]. In haematological patients, the increase of TTV load is closely associated with immune system activity and various infection indicators (such as ANC, CRP, PCT), consistent with previous research [41], indicating its potential as a biomarker for infection in haematological patients.

Multivariate analysis revealed a close association between TTV and lung infection, consistent with findings from a study on lung transplant recipients, possibly as an indirect result of immune response-induced granulocyte changes [42, 43]. In the analysis of TTV infection preference by age and season, we found a relatively higher detection rate of TTV in the 19–65 age group and significantly higher detection rates in winter for both haematological and non-haematological patients. This variation may be related to seasonal changes in patients’ immune status. In summary, our study elucidated the association between the most common virus, TTV, in haematological patients and their clinical characteristics and clinical detection parameters. These findings can assist clinicians in better understanding the role of TTV infection in the progression of haematological diseases. Further research is required to clarify the clinical significance of TTV and its interaction with the immune system.

Among RNA viral infections, the most common was the HPgV-C virus, with a significantly higher detection rate in patients who underwent haematopoietic stem cell transplant, regardless of granulocytopenia, consistent with earlier findings [44]. Previous studies have indicated the immunomodulatory effects of HPgV-C in patients with HIV and its frequent detection in transplant recipients, suggesting that the activation and persistent infection with HPgV-C may be primarily related to immune system disruption in patients with a mature immune system. Further investigation of HPgV-C revealed higher detection rates in relatively immunocompetent younger and middle-aged patients, whereas lower detection rates were observed in immunocompromised adolescent and older patients. No association was observed between HPgV-C and fever, or other clinical features or indicators. Therefore, clinical attention may not be required to address this issue. However, the relationship between HPgV-C infections and the immune system requires further investigation.

Members of the Herpesviridae family were most commonly detected in our analysis. Herpesviridae members, such as CMV and HHV-6, had significantly higher detection rates in non-granulocyte-deficient haematologic patients than in non-haematologic patients. This may be related to the fact that these viruses target human host blood cells, particularly monocytes and lymphocytes [38–40]. Most people may latent herpesvirus infections without apparent clinical symptoms, and haematological diseases may make it easier for these herpesviruses to replicate and cause infections in these patients [45, 46]. Notably, HSV1 was detected more frequently in patients with granulocyte-deficient haematology, possibly because HSV-1 typically resides in human neural cells and has a lower association with blood cells [47].

In the analysis of differences in detection rates based on clinical patient characteristics, we found that CMV, similar to TTV, had higher detection rates in the post-transplant population and in Branch Hospital 3, whereas non-viral pathogens showed the opposite trend. This suggests that post-transplantation CMV may play a role similar to that of TTV in modulating the immune response. In contrast, EBV infection was more common in the > 65 years age group and was higher in patients with haematologic diseases, especially in the 19–45 years age group [11, 48]. Moreover, EBV infection typically induces fever symptoms in patients. Additionally, we found that haematological patients were more prone to HHV-6B infection in 1–6 months after transplantation, which is consistent with previous research findings [49, 50]. In clinical practice, clinicians should be cognizant of the susceptibility of these members of the Herpesviridae family to different disease courses in haematological patients to achieve early prevention, thus helping reduce the incidence of infections in patients.

B19V and HPyV are also common viruses in haematologic patients [11, 12]. Our analysis revealed that B19V was more susceptible to non-granulocyte-deficient haematologic patients, post-transplantation, and during winter. Previous studies have indicated that B19V infection typically presents with symptoms such as fever, sore throat, headache, muscle pain, and rash. In immunocompromised individuals, it may lead to more severe conditions, including severe anaemia or organ damage [51, 52]. This suggests that clinicians should pay more attention to patients during these susceptible stages. Furthermore, in our multifactorial analysis, we found some previously unreported conclusions, such as the association between B19V and hyperlipidaemia (*P* < 0.05; Fig. 5a). However, the clinical significance and connections between these findings require further confirmation.

HPyV includes common variants such as HPyV-1, HPyV-2, and HPyV-5 [53]. Research indicates that in patients with haematological disorders, HPyV-2 infection can lead to bladder mucosal damage and symptoms of haemorrhagic cystitis, with a high detection rate observed in patients who underwent kidney transplant [9]. In the multivariate analysis, we observed a correlation between HPyV infection and solid tumours and urinary tract infections, consistent with previous studies [54]. Therefore, clinicians should be vigilant of HPyV infection when patients present with similar symptoms. Additionally, we observed a correlation between HPyV and lung infections, which may be an indirect effect of other diseases or complications. In addition to these highly detected viruses, we also detected other viruses such as adenovirus, HBV, HPV, EV41, and rubella virus. These viruses can also lead to severe complications in patients under certain conditions and require clinical attention [10–12, 55, 56].

However, this study has some limitations. Because our samples were obtained from clinical patients, some doctors did not suspect RNA virus infection based on the patient’s clinical symptoms; therefore, some samples only underwent DNA processing. In the experiments, we did not enrich the viruses in the samples to accommodate the detection of non-viral pathogens, which may have led to the under-detection of some viruses at low viral loads. Additionally, we were unable to assemble viral genomes for more in-depth analysis.

In the future, more detailed and in-depth research is required to explore the dynamic changes in these haematologic viruses during the development of haematologic diseases and their impact on disease progression. This will provide a more comprehensive understanding of the roles of these haematological viruses in the human body.

### Supplementary Information

Supplementary Material 1.

Supplementary Material 2.

## Data Availability

The sequence data, including all data with human sequences removed, have been deposited at the China National Center for Bioinformation under BioProject ID PRJCA026890.
