# Supplementary figures and images for: The plasma viral communities associate with clinical profiles in a large-scale haematological patients cohort
Source: Microbiome. 2024 Jul 23;12:137. doi: 10.1186/s40168-024-01855-4 (PMC11265361; doi:10.1186/s40168-024-01855-4)

# Non-hematological diseases

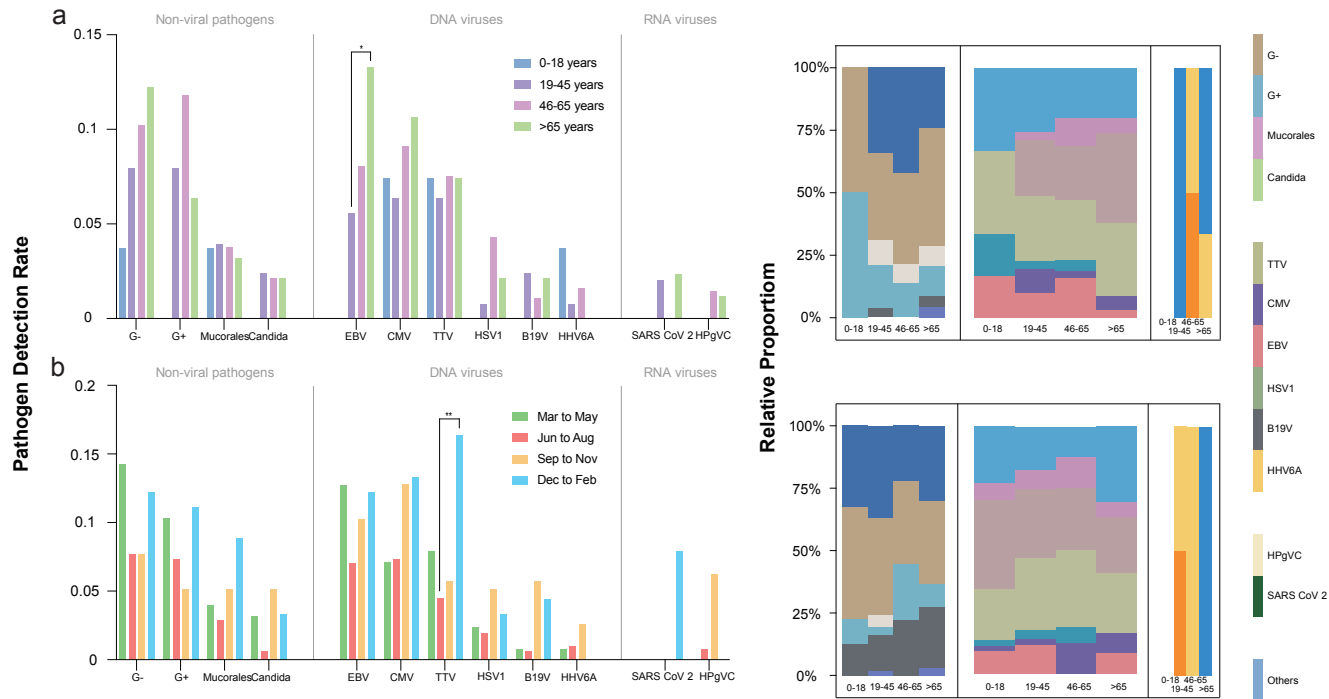

Supplement: Supplementary file 3 — Supplementary Material 2. [file 40168_2024_1855_MOESM2_ESM.pdf]
